# Retraction statement: Prenatal and neonatal complications of COVID‐19: A systematic review

**DOI:** 10.1002/hsr2.1451

**Published:** 2023-07-26

The following article, published online on {First published: 15 February 2022} in Wiley Online Library {https://onlinelibrary.wiley.com/doi/full/10.1002/hsr2.510} has been retracted by agreement between the journal's Editor‐in‐Chief, Esmaeil Mehraeen and John Wiley & Sons Ltd. The retraction has been agreed given the journal has received evidence confirming that the peer review process of this paper was manipulated. As a result, the conclusions reported in the article are not considered reliable.
